# Isotope Coded Protein Labeling analysis of plasma specimens from acute severe dengue fever patients

**DOI:** 10.1186/1477-5956-10-60

**Published:** 2012-10-26

**Authors:** Romain Fragnoud, Javier Yugueros-Marcos, Alexandre Pachot, Frederic Bedin

**Affiliations:** 1 BioMérieux SA, Chemin de l’Orme, 69280, Marcy l’Etoile, France

## Abstract

**Background:**

Dengue fever is the most important arthropod born viral disease of public health significance. Although most patients suffer only from flu-like symptoms, a small group of patient experiences more severe forms of the disease. To contribute to a better understanding of its pathogenesis this study aims to identify proteins differentially expressed in a pool of five viremic plasma from severe dengue patients relative to a pool of five non-severe dengue patients.

**Results:**

The use of Isotope Coded Protein Labeling (ICPL^TM^) to analyze plasma depleted of twenty high-abundance proteins allowed for the identification of 51 differentially expressed proteins, which were characterized by mass spectrometry. Using quantitative ELISA, three of these proteins (Leucine-rich glycoprotein 1, Vitamin D binding-protein and Ferritin) were confirmed as having an increased expression in a panel of severe dengue plasma. The proteins identified as overexpressed by ICPL^TM^ in severe dengue plasma involve in clear up action after cell injury, tissue coherence and immune defense.

**Conclusion:**

This ICPL^TM^ study evaluating differences between acute severe dengue plasmas and acute non-severe dengue plasmas suggests that the three proteins identified are overexpressed early in the course of the disease. Their possible use as biomarkers for the prognostic of disease severity is discussed.

## Background

Dengue virus (DV) is a tropical mosquito-born flavivirus (family *Flaviviridae*) which infects an estimated 50 to 100 million people per year in tropical and subtropical areas. After an incubation period of 3–7 days, symptoms start suddenly and follow three phases: the febrile/acute phase, the critical phase and the recovery phase. Clinical manifestations of the febrile phase include biphasic fever, body pain, maculopapular rash and minor hemorrhages [1,2]. For a small proportion of patients, essentially children and young adults, the critical phase corresponds to the appearance of more severe symptoms, the dengue hemorrhagic fever (DHF), with or without Dengue shock syndrome (DSS). The pathogenesis of DHF/DSS has not been fully elucidated although several hypotheses have been proposed [3].

Immune components, and in particular antibodies, have been associated with pathogenicity through several mechanisms. Antibody-dependent enhancement of a secondary infection, immune complex formation and complement activation, cross-reactivity with proteins such as endothelial cells or coagulation proteins, inflammatory activation or apoptosis are the mechanisms proposed for antibody-mediated immunopathogenesis [4-7]. Moreover, several authors have reported that antibodies also play a central role in allergic processes leading to hemorrhagic syndrome [8,9].

Other immune components including memory T-cells, innate immunity effectors and complement factors have been shown to modulate the outcome as well [10,11]. The dengue non structural protein 1 (NS1) can also participate to the pathogenicity in cooperation with the complement proteins [10].

No specific antiviral treatment for DF and DHF exists at the moment. Available therapies are symptomatic and are administered to correct and control the clinical manifestations of hemorrhages and shock. Early diagnosis of virus infection and successful prognosis of DF complications are essential to adjust patient management. Commonly used laboratory diagnosis methods consist of virus isolation, viral nucleic acid amplification, detection of viral antigen (NS1) or detection of anti-dengue antibodies (IgG and/or IgM). No test to monitor and predict disease severity and outcome is currently available.

The identification of proteins specifically present in plasma before DHF/DSS symptoms appearance may lead to the development of new biomarkers for prognosis. Quantitative or semi-quantitative proteomic approaches such as SILAC (Stable-Isotope Labeling by Amino acids in Cell culture) or ICPL^TM^ (Isotope Coded Protein Labeling ^TM^) are potentially useful to find pattern of protein markers differentially expressed for a given disease [12,13].

In the present study, ICPL^TM^ has been applied to profile the protein expression differences existing between non-severe (DF) and severe dengue (SD) plasma samples taken early in the course of the disease. Quantitative ELISA were performed to confirm the proteomic data. The identification of these markers might contribute to improve prognosis of severe dengue and also patient care and management.

## Results

### Preparation and controls of sample pools for ICPL^TM^

Two groups of five plasma specimens (28 μL each; total volume of 140 μL), representative either of DF or SD, were constituted. The composition of each group is summarized in Table 1. All plasma came from subjects identified as having a secondary infection to dengue virus serotype 2 (DV2) and were taken between the onset and the defervescence, which corresponds to the beginning of the critical phase. The criteria used to determine that the infections were secondary were both based on the patient medical record and, when possible, on the immunoglobulin M (IgM)/IgG specific level. All samples were both positive for the presence of virus and for the presence of NS1 protein, indicating they were taken during the acute phase of the disease. For some of the plasma samples, only a low level of IgM was found. The sex ratio (M/F=3/2), the age (9–14 years old), and the collection day after onset (2–5 days) were similar between the two groups. All the SD patients were hospitalized and presented signs of plasma leakage and hemorrhages [3]. No comorbidity was reported.

**Table 1:** Characteristics of plasma pools used in ICPL™

**Pool**	**Age (year)**	**Days after onset**	**Sex**^**a**^	**DV2 virus**^**b**^	**DV2 IgM**^**c**^	**NS1**^**d**^	**Secondary infection**
SD	10	4	F	Pos.	W	Pos.	Yes
	12	3	F	Pos.	W	Pos.	Yes
	13	2	M	Pos.	N	Pos.	Yes
	11	2	M	Pos.	N	Pos.	Yes
	14	5	M	Pos.	NT	Pos.	Yes
DF	9	3	F	Pos.	N	Pos.	Yes
	13	3	F	Pos.	W	Pos.	Yes
	10	3	M	Pos.	N	Pos.	Yes
	9	4	M	Pos.	W	Pos.	Yes
	12	5	M	Pos.	W	Pos.	Yes

(a) Gender. F : female, M : male. (b) Pos. : DV2 detected in sample. (c) Level of specific IgM in sample. W : weak, N : not detected, NT : not tested. (d) Pos.: NS1 detected in sample.

After depletion of the twenty most abundant proteins using immunodepletion columns (Proteoprep20, Sigma), each pool was controlled on polyacrylamide gel after an EZBlue dying. As an example, the depletion of the serum albumin was estimated around 98% (data not shown).

### Identification of differentially expressed proteins by ICPL^TM^ and MS/MS

In ICPL analysis, the relative expression is measured using the H/L ratio which corresponds to the global average ratio of peptides labelled with heavy (H) or light (L) reagent, as explained in the Material and Methods section. Among the 124 proteins identified by mass spectrometry after ICPL^TM^ experiment, only 51 had a H/L ratio calculated with a minimum of two peptides. For another group of 18 proteins, the H/L ratio was calculated with an unique peptide. For these proteins the results were considered as not significant. For a third group of 55 proteins, no information on the relative quantity was available. These two last groups were consequently excluded from subsequent analysis.

The results obtained for the 51 proteins with reliable data are summarized on the Table 2. An average H/L ratio (cf. Average column) higher than 1 meant that the signal obtained on SD pool was higher than the signal monitored for the DF pool. A ratio lower than 1 meant that the signal was higher for DF samples. The mean (m) of all these ratios and the corresponding standard deviation (s) were 1.4 and 0.83, respectively. Only four proteins (Leucine-rich alpha-2 glycoprotein 1, Galectin 3 Binding-Protein, C-Reactive Protein and the Ferritin Light-chain) had an average H/L ratio higher than 2.23, corresponding to (m+1 s). However, in order to increase the probability to find potential biomarkers for severe dengue, we choose to include three additional proteins (Vitamin D Binding-Protein, Afamin and Fibronectin). For these proteins, the average ratio was higher than 1.6. The Ferritin Light chain with an average ratio of 5.75 had the highest value. Quite the opposite, three proteins, Peroxiredoxin-2, Haptoglobin and Complement component C7 had an average ratio lower than 0.57, corresponding to (m-1 s). Haptoglobin and Complement component C7 were two contaminant proteins, imperfectly removed by the kit of depletion. Thus, the Peroxiredoxin-2 was the unique protein which had a ratio lower than 0.57. Finally, the selected proteins were Peroxyredoxin-2, Vitamin D Binding-Protein (VitDBP), Afamin, Fibronectin, Leucine-rich alpha-2 glycoprotein 1 (LRG1), Galectin 3 Binding-Protein, C-Reactive Protein and Ferritin Light-chain. These proteins, with an average ratio higher than 1.6 or lower than 0.57, represented 20% of the 51 selected proteins. The Table 2 also gave the standard deviation (cf. Max. average column). This standard deviation corresponded to a percentage generally included between 10 and 30% of the average ratio, even though sometimes it was higher: 35% for Haptoglobin, 40-45% for Apolipoprotein A, VitDBP and LRG1, or more for Complement factor H-related protein or Histidine-rich glycoprotein. The seven selected proteins were classified into several categories based both on their differential expression and on their functional significance (Table 3). They belong to a variety of functional categories (transport protein, inflammatory protein, adhesion, cell organisation and interaction). Interactions have been identified between G3BP and Peroxiredoxin 2 or LRG1 and Fibronectin using the STRING protein-protein interactions database ( http://string-db.org). All are soluble proteins either located in the cytosol or secreted.

**Table 2:** Host proteins identified and relative quantification by ICPL™

**Protein names and species**	**Accession number**	**H/L**^**a**^	**Average**^**b**^	**Max. Average**^**c**^
*Peroxyredoxin 2; Homo sapiens*	*P322119*	*9*	*0,33*	*0,08*
Haptoglobin; Homo sapiens	P00738	13	0,43	0,150
complement C7; Homo sapiens	P10643	8	0,48	0,030
Apolipoprotein A; Homo sapiens	P06727	3	0,68	0,310
Fibrinogen Alfa chain Homo sapiens	P02671	4	0,79	0,030
Apolipoprotein E; Homo sapiens	P02649	6	0,8	0,0140
Serum amyloïd A Protein; Homo sapiens	P02735	7	0,92	0,070
Fibrinogen beta chain Homo sapiens	P002675	9	0,93	0,050
Inter alpha-trypsin inhibitor H1 Homo sapiens	P19827	7	0,94	0,230
Complement facteur H protéine; Homo sapiens	Q03591	14	0,95	0,140
Inter alpha-trypsin inhibitor H2; Homo sapiens	P19823	15	0,98	0,27
Fibrinogen gamma chain; Homo sapiens	P02679	3	0,99	0,01
Vitronectin; Homo sapiens	P04004	2	1	0,1
Complement facteur B; Homo sapiens	P00751	8	1,06	0,1
Complement C4; Homo sapiens	POCOL4	3	1,06	0,304
Serum Albumin; Homo sapiens	P02768	27	1,09	0,18
alpha-2 antiplasmin; Homo sapiens	P08697	5	1,18	0,203
Antithrombin-III; Homo sapiens	P01008	16	1,23	0,13
Apolipoprotein-A1; Homo sapiens	P02647	74	1,24	0,21
Retinol binding-protein 4; Homo sapiens	P02753	5	1,24	0,13
Prothrombin; Homo sapiens	P00734	4	1,26	0,15
Complement factor 1; Homo sapiens	P05156	3	1,27	0,02
Beta-2 microglobulin; Homo sapiens	P61769	3	1,27	0,05
Ig alpha-1 chain C Homo; sapiens	P01876	2	1,27	0,042
Hemopexin; Homo sapiens	P02790	28	1,27	0,14
Alpha-1B glycoprotein; Homo sapiens	P04217	4	1,28	0,08
Serum Amiloid A4 protein; Homo sapiens	P35542	6	1,29	0,14
Kininogen-1; Homo sapiens	P01042	3	1,3	0,346
Ig Mu chain C; Homo sapiens	P01871	2	1,3	0,057
Cysteine-rich secreted protein 2; Homo sapiens	P16562	2	1,3	0,212
Zinc-alpha-2 glycoprotein; Homo sapiens	P25311	7	1,3	0,12
Extracellular matrix protein 1 Homo sapiens	Q16610	2	1,32	0,057
Pigment epithelium-derived factor; Homo sapiens	P36955	3	1,35	0,1
Complement C3; Homo sapiens	P01024	7	1,37	0,19
Attractin; Homo sapiens	O75882	4	1,38	0,2
Complement-facteur H-related protein 2; Homo sapiens	P36980	2	1,41	1,443
Apolipoprotein B-100; Homo sapiens	P04114	6	1,43	0,26
Complement facteur H; Homo sapiens	P08603	24	1,46	0,41
Alpha-1 antichemoptrypsin; Homo sapiens	P01011	6	1,48	0,2
Angiotensinogen; Homo sapiens	P01019	4	1,54	0,25
Histidin-rich glycoprotein; Homo sapiens	P04196	2	1,54	1,259
Inter-alpha trypsin inhibitor chain H4; Homo sapiens	Q14624	5	1,54	0,09
Serum-amyloïd P-component; Homo sapiens	P02743	15	1,56	0,23
Clusterin; Homo sapiens	P10909	7	1,57	0,25
*Vitamine D Binding-protein; Homo sapiens*	*P02774*	*13*	*1,63*	*0,75*
*Afamin; Homo sapiens*	*P43652*	*6*	*1,85*	*0,34*
*Fibronectin; Homo sapiens*	*P02751*	*9*	*1,99*	*0,28*
*Leucine-rich alpha-2 glycoprotein 1; Homo sapiens*	*P02750*	*2*	*2,26*	*0,884*
*Galectin-3 binding-protein; Homo sapiens*	*Q08380*	*5*	*2,66*	*0,26*
*C-reactive protein; Homo sapiens*	*P02741*	*3*	*3,87*	*0,365*
*Ferritin light chain; Homo sapiens*	*P02792*	*5*	*5,75*	*1,7*

(a) Number of heavy and light peptide identified for a protein. (b) Global average of the H/L ratio. (c) H/L ratio standard deviation. Lines in italic indicate the selected proteins.

**Table 3:** Functional classification of the proteins selected after ICPL™

**Over represented in DF pool**	**Over represented in SD pool**	**Functional category**	**Subcellular location**	**Comments**
Peroxiredoxin 2	-	Oxidative stress regulatory protein	Cytosol	Interacts with G3BP
**-**	Galectin-3 Binding Protein (G3BP)	cell-cell and cell-matrix interactions; immune response (natural killer and cytotoxicity)	Secreted	Interacts with Peroxiredoxin 2
**-**	Vitamin D Binding-Protein	Multifunctional; Vitamin D transportation (albumin protein familly)	Secreted	-
**-**	Afamin	Multifunctional; Vitamin E transportation (albumin protein family)	Secreted	-
**-**	Fibronectin	Cell migration, adhesion and matrix organization	Secreted	Interacts with LRG1
**-**	Leucine-Rich Glycoprotein 1(LRG1)	protein-protein interaction, signal transduction, and cell adhesion and development	Secreted	Interacts with Fibronectin
**-**	C-Reactive Protein	Response to inflammation	Secreted	-
**-**	Ferritin LC	Iron storage and release	Cytosol	-

Despite depletion of the twenty most abundant proteins, Fibrinogen, Serum Albumin, Immunoglobulins and Complement proteins were also identified. However, for these proteins the H/L average ratio was not considered as significant.

### Validation of differentially expressed proteins by quantitative ELISA

To confirm the ICPL^TM^ data, specific ELISA were performed using individual plasma samples from SD or DF patients. For each pathology, between 12 and 20 specimens were tested. Testing was also performed on three healthy plasmas (not dengue virus-infected). All dengue samples corresponded to secondary dengue infections and had been taken between onset and defervescence (between days 2 and 5) from patients infected with various DV serotypes (DV1, DV2 and DV3). The patients were between 4 and 16 years old. The M/F sex ratios for DF and SD samples were 5/12 and 7/16 respectively. These samples were both controlled positive for the presence of virus by RT-PCR and for the NS1 protein by a commercial ELISA (NS1 Platelia^TM^). The SD patients were hospitalized and presented signs of plasma leakage and hemorrhages. No comorbidity was reported.

**Figure 1:**
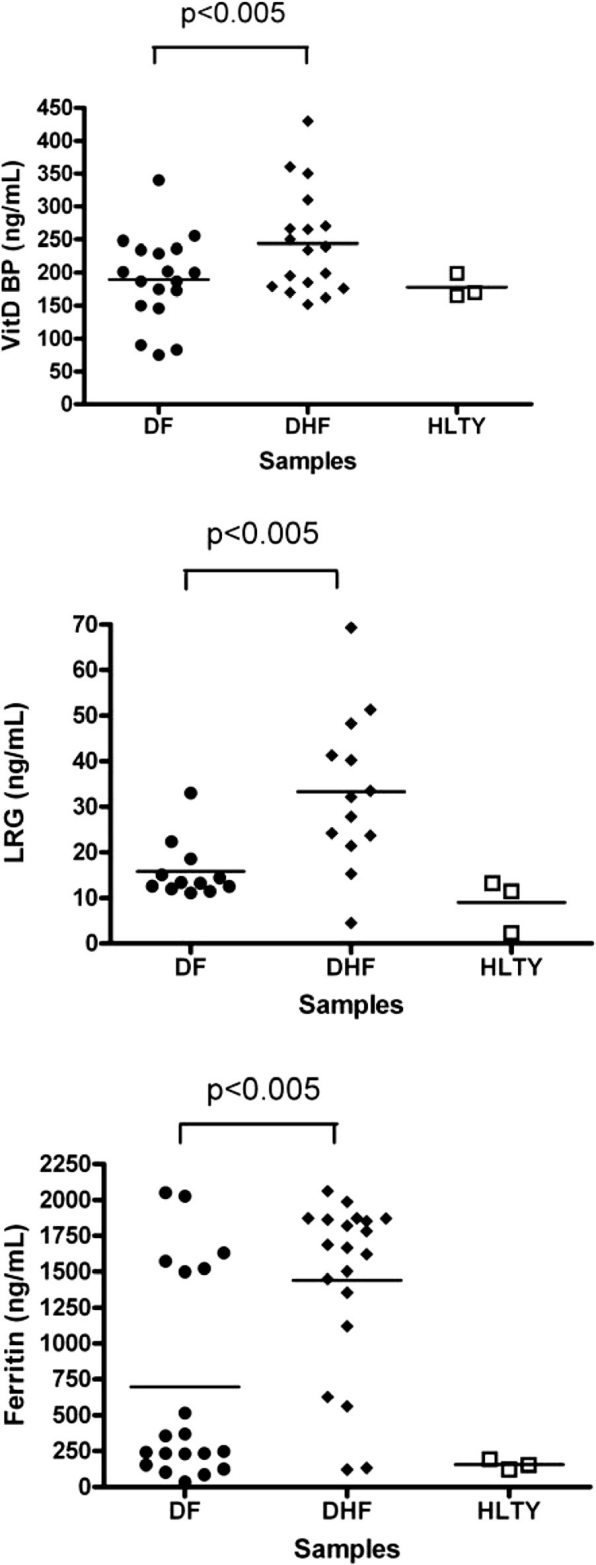
**Comparison of protein concentration detected by quantitative ELISA.** Acute plasma from classical dengue patient (DF), acute plasma from severe dengue patients (DHF) or healthy plasma from non-dengue patients (HLTY) were tested by ELISA for **(a)** VitDBP, **(b)** LRG1 and **(c)** Ferritin. Each value corresponds to the mean of two experiments in duplicate.

For Peroxiredoxin-2 detection, a specific ELISA was set-up in-house. Six commercial quantitative ELISA were used for the other proteins. For Peroxiredoxin, Afamin, Galectin-3 Binding Protein and C-Reactive Protein, no significant difference was found between DF and SD samples (data not shown). For VitDBP, Ferritin, LRG1, the results were illustrated in the Figure 1. These results showed that for these proteins a difference in the expression level existed and was significantly higher in SD patients.

## Discussion

Dengue is an important health problem in tropical and sub-tropical areas. Although the pathogenesis of the disease has been studied intensively, some aspects remain not well understood. Genome-wide expression profile analysis on microarrays or 2D-electrophoresis protein difference analysis in combination with MALDI-TOF/TOF Mass Spectrometry have been used to search for biomarkers that could serve as prognostic tools or therapeutic targets for severe dengue. Proteins belonging to the complement pathway or involving in processes such as inflammation, signal transduction or translation/transcription have been identified but there is no clear consensus on their potential use in the severity prognostic [14-17]. More recently, Brasier et al. [18] described a new pre-fractionation method on acute dengue Venezuelan plasmas followed by a 2D-gel electrophoresis in tandem with LC-MS/MS and a non-parametric analysis. Previously unidentified biomarkers having biological significance in process underlying severe dengue such as capillary leakage or hepatic injury, have been found.

The plasma, which corresponds to the unclotted blood after removal of cellular components, has constant intimacy with different body parts and contains proteins released by diseased tissues [19]. The comparison of circulating proteins in plasma from patients developing different aspects of a same disease may lead to the discovery of biomarkers for diagnosis, prognosis and disease monitoring [20]. The present study aims to identify differences in circulating proteins during different degrees of severity of dengue virus acute infections.

Stable isotope protein labeling combined with Mass Spectrometry is a powerful tool to identify and relatively quantify proteins within complex protein mixtures such as tissue extracts or body fluids. The ICPL^TM^ technique allows high-throughput quantitative proteome profiling on a global scale. It is used for protein profile comparison [21,22].

Here, ICPL^TM^ was used to compare mild and severe dengue plasmas for the identification of differentially expressed proteins. To increase the chance of finding low-abundance proteins the study was conducted on plasma depleted of the twenty most abundant proteins. For this purpose, it has previously been proven that immune-capture columns are useful [23]. However, the higher the number of depleted proteins is, the more important the drawback in removing associated proteins and peptides is. This drawback could have an impact on the list of identified markers of severity [23].

For each pathology, the use of pool of five plasma specimen homogeneous in age, sex ratio, days of collect after onset of symptoms and serotype would limit the ICPL identification of idiotypic markers specific of each individual plasma sample. It would also promote the discovery of markers that should be common to all samples, such as severe dengue markers. Although sometimes criticized [24], the use of a pool instead of several separate samples for proteomic analysis has been reported in recent studies [25,26].

Three proteins, identified by mass spectrometry, were confirmed by ELISA to have significant higher expression levels in SD plasmas. These proteins were Leucine-Rich alpha-2 Glycoprotein 1, Ferritin and Vitamin D Binding-Protein.

The physiological function of LRG1 is still unknown. In 2002, O'Donnell et al. [27] have suggested a physiological role in neutrophilic granulocytes. In 2006, Cummings et al. [28] identified LRG1 as cytochrome c inhibitor in sera*.* More recently, Codina et al. [29] have shown that LRG1 protects against Cyt*c*-induced lymphotoxicity. LRG1 has also been shown to be involved in protein-protein interaction, signal transduction, cell adhesion and development. LRG1 was identified as a serum biomarker that identifies patients with heart failure [30]. In the literature, LRG1 has never been associated to the dengue severity. Interestingly, LRG1 has been shown to interact with the Fibronectin also identified in the present study as being over-expressed in SD plasmas [31].

VitDBP is a 52 kDa protein that binds to vitamin D as well as its plasma metabolites and transports them to target tissues. VitDBP binds monomeric actin in addition to Vitamin D. The protein forms three domains. The structure of VitDBP is similar to the Human Serum Albumin [32,33]. It is presumed that the function of VitDBP binding actin is to clear up any actin that enters the blood stream as a result of cell injury. Its affinity for actin monomers is high [34] and binding involves residues from all three domains [32,33]. VitDBP also participates to macrophage activation and chemotaxis. The protein has been already associated to the pathogenesis of dengue by a Two-dimensional difference gel electrophoresis (DiGE) analysis on Brazilian plasma specimens [14].

Ferritin is a globular protein which serves to store iron in a non-toxic form, and to transport it to areas where it is required [35]. Free iron is toxic to cells because it catalyzes the formation of free radicals from reactive oxygen species [36]. Within cells, iron is stored in a protein complex as ferritin or hemosiderin. Ferritin concentrations increase drastically in the presence of an infection or with cancer [37]. The inflammatory response may cause ferritin to migrate from the plasma to the cells in order to deny iron to the infectious agent [37]. The concentration of ferritin has also been shown to increase in response to stresses such as anoxia [38]. In a previous study, high serum ferritin levels have been demonstrated by ELISA to be a biomarker of dengue hemorrhagic fever in Thai children [39]. Interestingly, proteins involved in inflammatory process have often been identified as potential markers of dengue severity [14-17].

All these proteins are related to several functions: transportation, adhesion and iron storage and seem to participate in the preservation of the body homeostasis during dengue infection.

## Conclusions

This is the first report of a study using ICPL^TM^ to evaluate differences between acute severe dengue plasma and acute non-severe dengue plasma. Among 8 proteins found having significant differential expression, we confirmed by ELISA that 3 proteins (Leucine-Rich alpha-2 Glycoprotein 1, Ferritin and Vitamin D Binding-Protein) were present at higher concentrations in SD samples early in the course of the disease. The contribution of these proteins to disease evolution and final outcome needs to be further investigated. Interestingly and more importantly the study suggests that these proteins could be used as potential markers for dengue severity and could be part of a serological assay as an aid for better patient stratification and disease management. Moreover, it will be necessary to validate these biomarkers with samples from other cohorts coming from different parts of the globe where dengue is endemic.

## Methods

### Plasma specimens

Plasma samples from secondary dengue-virus serotype 2 (DV2) infection were obtained from the Institut Louis Malardé, Papeete, Tahiti (French Polynesia). These samples were collected from patients being part of retrospective studies reviewed and approved by the local Medical Ethic Committee in compliance with the ethical standards of the Declaration of Helsinki. Alternatively, other plasma samples of mixed serotypes (DV1, DV2 and DV3) coming from the same retrospective studies, were used in ELISA. All the samples were taken between the onset of symptoms and the defervescence. Dengue-negative plasma specimens were obtained from healthy donors through the National French Bloodbank (Etablissement Français du Sang, Lyon, France). Dengue samples were tested for the presence of the viral NS1 protein with the Platelia^TM^ ELISA following the manufacturer’s instructions (BioRad, Marnes-la-Coquette, France).

### Depletion of high-abundance plasma proteins

The twenty most abundant plasma protein were removed from 140 μL (5 plasmas × 28 μL) of the plasma pools using ProteoPrep20 Plasma immunodepletion Kit (Sigma Aldrich, Saint-Louis, USA) as recommended by the manufacturer. After filtration on a 0,2 μM device, the total proteins were quantified using the protein assay (Bio-Rad, Marnes-la-Coquette, France).

### ICPL^TM^ and protein identification by mass spectrometry

The ICPL^TM^ technique allows the comparative analysis of complex samples by an isotopic labeling of the intact proteins. ICPL^TM^ and protein identification have been conducted by InnovaProteomic (Rennes, France). Before ICPL^TM^-labeling, acetone precipitation of proteins from either DF or DHF depleted pool of plasma was carried out. To one volume of sample 5 volumes of acetone cooled to −20°C were added and incubated overnight at −20°C. Precipitated proteins were pelleted by centrifugation 15 min at 15,000 g at 4°C. Pellets were washed with cold 80% (v/v) acetone and dried. The protein concentration, previously determined by a Bradford assay, was used to adjust sample concentration at 3 mg/ml in ICPL^TM^ lysis buffer containing 6 M Guanidine-HCl pH8.5 (SERVA, ICPL^TM^ Kit). For equal amounts of proteins (60 μg for each sample), cysteines were alkylated by addition of 0.4 M of iodoacetamide and differentially labeled using the ICPL^TM^ kit containing ^12^C- (light labeling) and ^13^C_6_–Nicotinoyloxysuccinimide (heavy labeling) from Serva Electrophoresis GmbH (Heidelberg, Germany) as described by the manufacturer [40]. Thereafter the two ICPL^TM^ labeled samples were combined and proteins were precipitated by acetone to remove excess labeling reagent. Proteins were solubilized in Laemmli sample buffer, separated on 12% SDS-PAGE (GeBa, Interchim) in duplicate and stained with EZblue (Sigma Aldrich, Saint Louis, USA). After staining, the gel lanes were cut into 20 pieces and in-gel digestion by Trypsin was performed overnight at 37°C, according to the conventional method [41]. Finally, the peptides were extracted with 50% (v/v) acetonitrile containing 0.1% of formic acid [42]. The extracts were evaporated using a vacuum concentrator. The dried peptide samples were stored at −80°C until analysis.

Peptide separation was done by liquid chromatography using a nanoLC Ultimate system (Dionex GmbH, Idstein, Germany) coupled to an ion trap Esquire HCT ultra (Bruker Daltonics GmbH, Bremen, Germany). Mass spectra were recorded and the quantification was operated by the WARP-LC 1.1 software. To identify proteins, searches against the MSDB database (version 2007, 148 210 sequences for human) using Mascot 2.3 software (Matrix Science, in-house server) were carried out via Biotools software (Bruckner Daltonic). Proteins were identified with a score higher than 36 (p<0.05).

In order to eliminate false matches and incorrect protein identification, search on the SwissProt-Trembl_Decoy database was performed using the Mascot 2.3 software. The IRMa software [43] was used to filter the results so that the rate of false positives was lower than 1%.

The change in protein abundance was expressed by the calculation of a ratio between the two type of labeled peptides (heavy and light). To calculate the heavy/light (H/L) ratios of a protein at least two unique peptides labeled with heavy and light reagents were chosen respectively and a global average ratio was determined. For each result, standard deviation was calculated.

Three independent analytical replicates were performed for each 1D-gel band.

### Quantitative ELISA

Protein quantities were measured twice in duplicate on each DF or SD plasma specimens using commercial ELISA. Human Ferritin and LRG1 kits were purchased from IBL international (Hamburg, Germany) and the Human Vitamin D binding–protein from USCN Inc. (Wuhan, China). Protocols were performed as recommended by the manufacturers. For each test a standard curve was established with serial dilution of calibrator in order to determine the protein concentration. Statistical analysis were performed using the Student *t* test and the GraphPadPrism 4.03 software.

## Competing interests

The authors declare to be employed by bioMérieux SA.

## Authors’ contributions

Conception and design of the experiments: RF, FB. Realization of the experiments : RF, FB, Writing of the paper: RF, FB, JYM. Review of the paper: FB, JYM, AP. All authors read and approved the final manuscript.
